# Substance Use in Pregnant Women Using the Emergency Department: Undertested And Overlooked?

**DOI:** 10.5811/westjem.2018.1.35630

**Published:** 2018-04-05

**Authors:** C. Leigh Moyer, Sean Johnson, Marilyn G. Klug, Larry Burd

**Affiliations:** University of North Dakota School of Medicine and Health Sciences, North Dakota Fetal Alcohol Syndrome Center, Department of Pediatrics, Grand Forks, North Dakota

## Abstract

**Introduction:**

The objective was to determine if pregnant women visiting the emergency department (ED) are tested for substance use as frequently as non-pregnant women.

**Methods:**

We captured all ED visits over a six-year period (2010–2016) from a single community hospital and identified women of childbearing age, defined for our study as 11–50 years old. We collected demographic data including age in years, ethnicity, body mass index, marital status, disposition, last encounter department, method of arrival, and day of week. An independent binary variable was created based on whether the woman was tested for alcohol or drugs (amphetamines, barbiturates, benzodiazepines, cannabis, cocaine, opioids) during her visit. We then compared rates of testing for substance use by pregnancy status.

**Results:**

We identified 61,222 ED visits by women of childbearing age (range 11–50, mean 30.5, standard deviation 9.6) over a six-year period from 2010–2016. Of the 57,360 non-pregnant women, 4.14% were tested compared to 1.04% of the 3,862 pregnant women tested with a relative risk of 0.25 (*p*<0.001, 95% confidence interval [CI] [0.183–0.341]). The most highly tested chief complaints for all women – psychiatric or substance use concerns – showed pregnant women were still 37% and 54% less likely to be tested, respectively (risk ratio [RR] 0.46, 95% CI [0.19–1.13]; RR 0.63, 95% CI [0.41–0.96]). Beyond pregnancy status, we found no significant interaction between patient demographics and substance use testing.

**Conclusion:**

Pregnant women presenting to the ED were 75% less likely to be tested for drug or alcohol use than non-pregnant women. Our study showed only pregnancy status as a statistically significant variable in drug- and alcohol-screening rates when pregnant and non-pregnant patient chief complaints and demographics were compared. Increased attention to the screening of pregnant women for substance use may be necessary to provide adequate care and intervention to this population.

## INTRODUCTION

Alcohol and drug use among women of childbearing age represents an increasing burden to society and healthcare providers across the United States. Substance use during pregnancy is associated with increased rates of obstetric complications, fewer prenatal visits, and poor perinatal outcomes.^1^–^4^ Fetal alcohol spectrum disorder (FASD), a serious consequence of prenatal alcohol exposure, is the leading preventable cause of birth defects and neurodevelopmental disability in the U.S. It often reoccurs within sibships and the mortality among birth mothers of children diagnosed with an FASD is increased by nearly 39-fold.^2^,^5^,^6^ Recent data demonstrate that 11.9% of non-pregnant women and 5.3% of pregnant women age 15–44 reported illicit drug use in November, 2016.^1^ Alcohol use at levels meeting criteria for binge or heavy drinking was reported by 23.7% of non-pregnant women and 2.8% of pregnant women.^1^

According to the National Institute on Drug Abuse, of the more than 130 million visits to emergency departments (ED) in 2009, 2.1 million (2.73%) were for drug abuse.^7^ From 2004 to 2009 ED visits for non-medical use of drugs increased 98% (nearly 20% per year), with 32% of patients reporting concurrent alcohol use.^7^ In 2005 the National Alcohol Survey found that 24% of individuals presenting to the ED reported high-risk drinking behaviors.^8^,^9^

Approximately 50% of pregnancies in the U.S. are unplanned, with fetal first trimester exposure rates of 56% for all women and 78.9% for women with recent alcohol dependence.^10^ While the majority of women cease or reduce alcohol consumption during pregnancy, in the U.S. alone every year around 80,000 women report drinking during all three trimesters.^11^ Women meeting criteria for a substance use disorder used ED services 57% more frequently than women who did not have a substance use disorder and were hospitalized 67 % more frequently.^1^ Given the high prevalence of substance use in the patient population most using ED services, this setting presents a unique opportunity to screen a high-risk population for substance use. However, limited data exist to examine the nuances of the screening process in the ED for substance use. In this study, we compared rates of screening for substance use among pregnant and non-pregnant women seeking care at an ED facility.

## METHODS

The project was approved by the Altru Health System Institutional Review Board and the University of North Dakota Institutional Review Board. We captured all ED records of women from a single community hospital for the years 2010 to 2016 (n=61,222). A woman was considered pregnant if her pregnancy status was recorded as “pregnant.” All other women were classified as not pregnant. Demographic data included age in years, race/ethnicity (White, American Indian/Alaskan Native, Hispanic, Black, and Other, which included Asian, Chinese, Filipino, Korean, Hawaiian, Pacific Islander, Nepalese, Samoan, Somalian, Vietnamese and unknown or refused), marital status (married, single, “Other,” which included life partner, significant other, fiancé, divorced, legally separated, widowed, other and unknown), and body mass index (BMI). We categorized data on how the woman arrived at the ED as either ambulatory or assisted (crutches/walker, wheelchair, cart/stretcher, or carried).

We created a dependent binary variable based on whether the woman was tested for alcohol or drugs during her ED visit or not tested. We examined the electronic medical record and the lab record for any test for substance use that was ordered or completed by the lab. Testing modalities included blood, urine, hair, or breathalyzer readings. We included testing for amphetamines, barbiturates, benzodiazepines, cannabis, cocaine, opioids, and alcohol.

**Population Health Research Capsule**

What do we already know about this issue?

In November 2016, 11.9% of non-pregnant women reported illicit drug use and 23.7% reported heavy drinking compared to pregnant women with 5.3% reporting drug use and 2.8% heavy drinking.

What was the research question?

Do emergency departments appropriately screen all women for substance abuse? Does pregnancy status modify screening rates?

What was the major finding of the study?

Pregnant women were screened for substance abuse only one-fourth as often as non-pregnant women.

How does this improve population health?

Improving screening for pregnant women may improve detection of substance abuse and allow for intervention during pregnancy and reduce risk for exposure during the next pregnancy.

The 399 unique chief complaint ICD-9 codes were then further grouped into 20 categories (Table 2). The last department used by the woman was categorized as ED, urgent care, or other (which included cardiac, ICU, obstetrics, oncology, orthopedics, surgical, and women’s and children’s units). Their dispositions were combined into two groups, internal (admitted, sent to labor and delivery, psychiatric care or transferred) or external (deceased, discharged, left against medical advice before or after triage, or referred to observation).

### Statistical Analysis

We compared the association between pregnancy status and drug/alcohol testing using the chi-square statistic with relative risk and 95% confidence intervals (CI). This association was assessed for the demographic covariates. The risk of drug/alcohol testing for pregnant woman relative to non-pregnant women was produced for levels of other variables using relative risk and 95% CIs. We tested interactions between pregnancy status and the demographic variables using the Breslow-Day test for homogeneity in odds ratios. Logistic regression was then used to test for interactions between pregnancy status and demographics or ED visit characteristics. We used SAS version 9.4 for analyses.

## RESULTS

We identified 61,222 visits by women age 11–50 years over the time period of 2010 to 2016. Table 1 summarizes the study subjects’ demographic information. The mean age was 30.5 (standard deviation [SD] 9.6 years) ranging from 11–50. We located data on BMI for 26,177 women with a mean value of 30.4 (SD 8.6) ranging from 11.5 to 85.9. The majority of women (78.5%) were White, 12% were American Indian /Alaska Native, 4.3% were Hispanic, and 2.4% were Black /African American. In the sample 3,862 (6.7%) were reported to be pregnant and 57,360 (93.3%) were not pregnant. Based on recorded pregnancy status, 4.14% of the 57,360 non-pregnant women were tested for drug or alcohol use and 1.04% of the 3,862 pregnant women were tested. The relative risk (RR) of a pregnant woman being tested was one-fourth that of a non-pregnant woman (RR=0.25; 95% CI [0.18 to 0.34]; p<0.001).

To check for effects of other variables that might have influenced this risk, we first tested the data using demographic variables age, BMI, race, marital status, and day of the week. While there was some variation, we found no significant interaction between the demographics and pregnancy associated with testing (Table 1). The RR for being tested was somewhat lower for single women, Whites, and on weekends and Thursdays. For both populations, not being married increased the risk of being tested but this was significant only for non-pregnant women (odds ratio=1.6) (95% CI [1.4 to 1.9]). We examined day of the week because in our community substance use increases on weekends.

Psychiatric concerns and substance use were the two most commonly tested presenting complaints (Table 2). Pregnant women presenting with substance use had a risk of being tested that was just less than half that of a non-pregnant woman (RR=0.46) (95% CI [0.19 to 1.13]). Women who were pregnant and presented with psychiatric complaints had a risk two-thirds of non-pregnant women (RR=.63) (95% CI [0.41 to 0.96]). Additional data on differences between groups by presenting complaint is shown in Table 2. The patient’s final disposition status showed some influence on testing risk. Based on pregnancy status, 30.05% (n=1,376) of the 4,579 non-pregnant women who were admitted to the ED were tested for drugs or alcohol, while only 9.29% (n=21) of the 226 pregnant women were tested. Of the 50,413 non-pregnant women discharged, 1.43% (n=723) were tested for drugs or alcohol, while only 0.56% (n=18) of 3,243 pregnant women discharged were tested. Additional detail about patient disposition status is shown in Table 3.

## DISCUSSION

Our study found that pregnant women presenting to the ED were 75% less likely to be tested for substance use than non-pregnant women. Even among the most-tested presenting complaints for all women (psychiatric or substance use concerns), pregnant women were still 37%-54% less likely to be tested. These data may suggest a relatively lower index of suspicion for substance use among pregnant women seeking care in the ED. This difference by pregnancy status was present even for women with similar presenting complaints and demographics in the ED.

While no prophylactic treatment exists at this time, early screening and counseling is the best practice to support women in decreasing the risk of the serious consequences associated with prenatal substance exposure.^12^ Identification of prenatal alcohol exposure is of particular importance as the long-term implications of alcohol exposure for the fetus have shown significant consequences in comparison to other substance exposures.^3^,^13^–^15^ With fetal first-trimester alcohol exposure rates of 56% for all women and 78.9% for women with recent alcohol dependence,^10^ the ED presents a unique opportunity to address a population of women with a greater than average incidence of alcohol and drug use. The opportunity to provide education and intervention at this stage is especially compelling among women of low socioeconomic status who are seen in the ED more frequently than in primary practice.^16^ Women who received treatment based on positive drug/alcohol screening results have been shown to subsequently have fewer future ED visits, injuries and hospitalization.^4^,^17^

## LIMITATIONS

Extrapolation of the results of this study is limited due to the small sample of women – pregnant or non-pregnant – tested in the ED for drugs and alcohol. An unknown portion of women with substance use may have been identified by history or observation and were not in need of additional testing. Additionally, in accordance with regional demographics the sampled population is largely White/Caucasian, which may limit the generalizability of this data to other populations. The power to detect significant risk differences between the two groups (when psychiatric concerns were the presenting complaint) for this sample size was 0.72 and when substance use was the presenting complaint the power was 0.82. Power for other diagnoses (prevalences > 0% for both groups) was low and ranged from 0.07 to 0.26.

While substance use does not equate to substance abuse, any use of alcohol or illicit drugs during pregnancy is contraindicated. Although not a factor in these data, a negative test does not rule out substance use in all women. The rates of testing in other ED services across the U.S. may differ. The data we collected do not allow for determination as to why clinicians select non-pregnant women for substance use screening 75% more often than pregnant women. This is an area for further research.

## CONCLUSION

The use of alcohol, prescription drugs, and illicit drugs is an important and growing public health problem. The ED presents a unique opportunity to address, intervene, and offer education to a population of women with a greater than average incidence of alcohol and drug use. Our research shows that increased attention to substance use in ED settings is warranted and that pregnancy status should not allay clinician concerns about substance use.

## Figures and Tables

**Table 1:** Patient characteristics of women upon arrival to the emergency department.

Characteristic	Non-pregnant	Tested	%	Pregnant	Tested	%
Pregnancy status, n (%)	57,360	2,377	4.14	3,862	40	1.04
Age, n (mean)	54,983 (30.77)	2,377	31.58	3,822 (25.96)	40	27.25
BMI, n (mean)	23,113 (30.53)	1,613	28.71	1,367 (30.11)	24	27.22
Race, n (%)						
White	45,291	1,874	4.14	2,742	22	0.805
AI/AN	6,886	354	5.14	459	12	2.61
Hispanic	2,431	78	3.21	193	1	0.52
Black	1,283	31	2.42	203	5	2.46
Other	1,469	40	2.72	265	0	
Marital status, n (%)						
Married	19,249	532	2.76	1,830	15	0.82
Single	29,475	1,391	4.72	1,728	20	1.16
Other	8,636	454	5.26	304	5	1.64
Arrival, n (%)						
Ambulatory	51,210	1,360	2.16	3,505	22	0.63
Assisted	5,011	848	16.92	249	14	5.62

*BMI*, body mass index; *AI/AN*, American Indian/Alaska Native.

**Table 2:** Presenting complaints for 61,222 women attending the emergency department from 2010 to 2016. The women were grouped by pregnancy status (pregnant and non-pregnant) to compare proportions tested for substance use.

Presenting complaint	Non-pregnant	Tested	%	Pregnant	Tested	%
Psychiatric, n (%)	1744	848	48.62	49	15	30.61
Substance use, n (%)	695	563	81.01	8	3	37.50
Other	n	n	%	n	n	%
Gastrointestinal	11980	132	1.10	871	5	0.57
Musculoskeletal	11519	80	0.69	384	3	0.78
Neurologic	6347	318	5.01	407	6	1.47
Immunologic	3835	16	0.42	234	0	0
Trauma	3472	154	4.44	166	4	2.41
Cardiac	2421	85	3.51	94	0	0
Oral	2314	1	0.04	155	0	0
Respiratory	2195	41	1.87	134	1	0.75
Genitourinary	2089	7	0.34	93	0	0
Gynecologic	1290	2	0.16	592	0	0
Treatment	1575	27	1.71	61	0	0
Dermatologic	1348	4	0.30	81	0	0
Ear	1137	0	0	77	0	0
Ocular	773	0	0		35	0
Pregnancy	187	0	0	136	3	2.21
Hematologic	150	4	2.68	8	0	0
Endocrine	140	13	9.29	5	0	0
Miscellaneous	1660	76	4.58	88	0	0
NA	489	6	1.23	184	0	0

**Table 3:** Patient disposition from emergency department visits for 61,222 women seen from 2010 to 2016. The women were grouped by pregnancy status (pregnant and non-pregnant) to compare proportions tested for substance use.

Characteristic	Non-pregnant	Tested	%	Pregnant	Tested	%
Discharged, n	50,413	723	1.43	3,243	18	0.56
Admitted, n	4,579	1,376	30.05	226	21	9.29
Other	n	n	%	n	n	%
LWBS after triage	582	4	0.69	46	0	0
AMA	233	28	12.02	15	0	0
Refer to observation	207	50	24.15	7	0	0
Send to psych	171	122	71.35	1	0	0
Eloped	104	8	7.69	7	0	0
LWBS before triage	95	0	0	6	0	0
Transferred	81	37	45.68	3	1	33.33
Send to L&D	5	0	0	44	0	0
Deceased	5	0	0	0	0	0

*LWBS,* left without being seen; *AMA*, against medical advice; *L&D*, labor and delivery.
